# Exploring clinical, echocardiographic and molecular biomarkers to predict bronchopulmonary dysplasia

**DOI:** 10.1371/journal.pone.0213210

**Published:** 2019-03-06

**Authors:** Maria Alvarez-Fuente, Laura Moreno, Paloma Lopez-Ortego, Luis Arruza, Alejandro Avila-Alvarez, Marta Muro, Enrique Gutierrez, Carlos Zozaya, Gema Sanchez-Helguera, Dolores Elorza, Andrea Martinez-Ramas, Gema Villar, Carlos Labrandero, Lucia Martinez, Teresa Casado, Irene Cuadrado, Maria Jesus del Cerro

**Affiliations:** 1 Pediatric Cardiology Department, Ramón y Cajal University Hospital, Madrid, Spain; 2 Department of Pharmacology, School of Medicine, University Complutense of Madrid, Instituto de Investigación Sanitaria Gregorio Marañon (IiSGM), Ciber Enfermedades Respiratorias (CIBERES), Madrid, Spain; 3 Neonatology Department, La Paz University Hospital, Madrid, Spain; 4 Neonatology Department, Institute of the Child and Adolescent, Clínico San Carlos University Hospital–IdISSC, Madrid, Spain; 5 Department of Pediatrics, Complexo Hospitalario Universitario de A Coruña, A Coruña, Spain; 6 Neonatology Department, Puerta de Hierro University Hospital, Majadahonda, Madrid, Spain; 7 Public Health and Preventive Medicine Unit, School of Public Health- Instituto de Salud Carlos III, Madrid, Spain; 8 Clinical Analysis Department, Getafe University Hospital, Getafe, Madrid, Spain; 9 Neonatology Department, Getafe University Hospital, Getafe, Madrid, Spain; 10 Pediatric Cardiology Department, La Paz University Hospital, Madrid, Spain; Vanderbilt University Medical Center, UNITED STATES

## Abstract

**Introduction:**

Bronchopulmonary dysplasia (BPD) is the most common chronic lung disease in childhood, related to prematurity, and the most common cause of pulmonary hypertension (PH) secondary to pulmonary disease in children. Moderate and severe BPD have a worse outcome and relate more frequently with PH. The prediction of moderate or severe BPD development in extremely premature newborns is vital to implement preventive strategies. Starting with the hypothesis that molecular biomarkers were better than clinical and echocardiographic factors, this study aims to explore the ability of clinical, echocardiographic and analytical variables to predict moderate or severe BPD in a cohort of extremely preterm infants.

**Patients and methods:**

We designed a prospective longitudinal study, in which we followed a cohort of preterm newborns (gestational age <28 weeks and weight ≤ 1250 grams). In these newborns we recorded weekly clinical and echocardiographic variables as well as blood and tracheal aspirate samples, to analyze molecular biomarkers (IL-6, IL-1, IP10, uric acid, HGF, endothelin-1, VEGF, CCL5). Variables and samples were collected since birth up to week 36 (postmenstrual age), time-point at which the diagnosis of BPD is established.

**Results:**

We included 50 patients with a median gestational age of 26 weeks (IQR 25–27) and weight of 871 g (SD 161,0) (range 590-1200g). Three patients were excluded due to an early death. Thirty-five patients (74.5%) developed BPD (mild n = 14, moderate n = 15, severe n = 6). We performed a logistic regression in order to identify risk factors for moderate or severe BPD. We compared two predictive models, one with two variables (mechanical ventilation and inter-ventricular septum flattening), and another-one with an additional molecular biomarker (ET-1).

**Conclusions:**

The combination of clinical and echocardiographic variables is a valuable tool for determining the risk of BPD. We find the two variable model (mechanical ventilation and echocardiographic signs of PH) more practical for clinical and research purposes. Future research on BPD prediction should be oriented to explore the potential role of ET-1.

## Introduction

Bronchopulmonary dysplasia (BPD) is a severe chronic lung disease in childhood and one of the most frequent sequel of prematurity [1]. The prevalence of BPD in very low birth weight infants (VLBWI) ranges between 15% and 50%, and is inversely related to birth weight and gestational age (GA) [2, 3].

BPD pathogenesis has evolved in the past decades from a lung lesion secondary to mechanical ventilation and elevated oxygen concentrations to a multi-factorial disease in which prenatal and postnatal factors are involved [4, 5]. The lungs of very low birth weight infants (VLBWI), at birth, undergoe a developmental arrest with decreased alveolarization and vascular growth [6]. BPD is further complicated by pulmonary hypertension (PH) in 17–45% of patients, with recent evidence on the role of early PH as a marker of BPD development [6–8]. Moderate and severe BPD is associated with a worse outcome and with a higher rate of complications and PH [9].

BPD diagnosis is established at 36 weeks postmenstrual age (PMA) [10], which is too late to implement preventive strategies. Therefore, the search for predictive factors for early identification of infants at high risk of BPD is an important area of research. Considering that moderate and severe BPD carry a worse outcome and higher rate of PH [9], the identification of risk factors for these grades of BPD would be optimal. In order to initiate preventive strategies that can reduce the incidence of the worst grades of the disease.

Determining the risk of BPD provides prognostic information, identifies infants who could benefit from preventive strategies, and helps stratify infants for clinical trial enrollment. Diverse clinical risk factors (GA, birth weight, gender, patent ductus arteriosus (PDA), ventilator settings, etc.) and molecular biomarkers (inflammatory pathways and vascular growth factors) have been studied in preterm infants as potential risk factors for BPD development and many prediction models have been developed [11–16]. Despite these efforts, no reliable and reproducible risk stratification model has been found that could be easily applicable in neonatal intensive care units.

Starting with the hypothesis that molecular biomarkers were better than clinical and echocardiographic factors, this study aims to explore the ability of clinical, echocardiographic and analytical variables to predict moderate or severe BPD in a cohort of extremely preterm infants.

## Patients and methods

The study was approved by the ethical committee of all participating hospitals (Hospital La Paz, Hospital Clinico (14/545-E), Hospital de Getafe, Hospital Puerta de Hierro and Complejo Hospitalario de Coruña (2015/001)). The hospital responsible was La Paz with the following approval number PI-1774. Written consent was obtained from all participants´ parents.

We designed a prospective multi-center cohort study that took place in the previously mentioned five Spanish hospitals. Extremely low gestational age newborns (ELGAN) of less than 28 weeks of gestation and birth weight under 1250 g, admitted to the participating hospitals during the study period were consecutively and prospectively recruited and followed from birth until 36 weeks of PMA, a time-point at which the diagnosis and severity of BPD was determined. Exclusion criteria were major congenital malformation, neurological lesion and mother with HIV.

Clinical, echocardiographic and analytical variables were registered at different time points (postnatal days 1, 3, 7, 14, 21 and 28) and entered into an electronic online database (www.pulmescell.org).

To homogenize patient management, all participating centers applied the same standard care treatment protocols, although we are aware that there may be details that can vary from one center to another. Steroid administration protocol was the most important, due to the potential alteration of molecular biomarkers, which we aimed to analyze in this study. Therefore the protocol for administration of steroids was exactly the same in all hospitals, in which there were no variations among centers.

This study was part of a preliminary research performed before starting a clinical trial (phase I), which aims to explore safety of umbilical cord-derived mesenchymal stem cells (UC-MSC) in ELGAN. The aims of this preliminary biomarkers study were to find a combination of clinical, echocardiographic, and molecular biomarkers capable of selecting ELGAN at high risk of developing moderate or severe BPD. This information could be used in the design of trials testing preventive strategies, such as UC-MSC. We also aimed to explore the profile of the selected molecular biomarkers in a cohort of ELGAN, treated with standard therapies that could afterwards be compared to the biomarkers profile of the patients participating in the clinical trial, treated with UC-MSC.

### Clinical variables

A patient was considered to have BPD if he/she required supplemental oxygen for at least 28 days. Severity was established at 36 weeks PMA, according to the definition by Jobe and Bancalari (Table 1) [10]. We did not include an oxygen reduction test to define BPD because it was not a standard procedure in all participating hospitals.

**Table 1:** BPD classification [10].

Treatment with supplemental oxygen for at least 28 days		
	<32 weeks	>32 weeks
**Time point of severity assessment**	**36 weeks PMA or hospital discharge**	**56 days post-natal age or hospital discharge**
**Mild BPD**	**Room air**	**Room air**
**Moderate BPD**	**<0.3 oxygen**	**<0.3 oxygen**
**Severe BPD**	**>0.3 oxygen and/or respiratory support (IPPV or CPAP)**	**>0.3 oxygen and/or respiratory support (IPPV or CPAP)**

BPD: Bronchopulmonary dysplasia. PMA: post-menstrual age. IPPV intermittent positive-pressure ventilation. CPAP: continuous positive airway pressure.

### Echocardiographic variables and PH determination

A complete echocardiography was performed to all patients at inclusion (day 1), to rule out congenital heart disease. At days 3, 7, 14, 21 and 28, we performed a complete echocardiographic study searching for PDA, atrial septal defect, pulmonary vein stenosis and PH. PH was defined in three grades (Table 2), if the patient had findings from two different PH grades, the highest grade was always assigned. The echocardiography protocol was discussed and agreed upon by the pediatric cardiologists working in the 5 participating hospitals, in order to reduce variability among the echocardiographic variables. Each cardiologist from each center had an investigator´s handbook with the images of the more subjective parameters in order to minimize possible bias. Other parameters that can be quantified, such as right atrium and right ventricle dilation, were compared with Zscore.

**Table 2:** Echocardiographic pulmonary hypertension evaluation.

Grades	Echocardiographic features
**No PH**	IVS type I. RV pressure (measured by tricuspid regurgitation) <35% of systemic arterial pressure. Normal right heart dimensions.
**Mild PH**	IVS type I-II. RV pressure (measured by tricuspid regurgitation) 35–50% of systemic arterial pressure. Mild dilation of the right atrium and RV.
**Moderate PH**	IVS type II. RV pressure (measured by tricuspid regurgitation) 50–70% of systemic arterial pressure. Moderate dilation of the right atrium and RV.
**Severe PH**	IVS types II-III or III. RV pressure (measured by tricuspid regurgitation) >70% of systemic pressure. Severe dilation of the right atrium and RV.

PH = pulmonary hypertension, IVS = interventricular septum, RV = right ventricle.

### Analytical biomarker determination

Blood samples were collected at days 1, 3, 7, 14, 21 and 28. Tracheal aspirate fluid was also collected at the same time-points, but only in intubated patients. The levels of IL-6, IL-1B, IP-10, CCL5 and endothelin-1 (ET-1) were analyzed in plasma, and IL-6, IL-1B, IP-10, CCL5 and uric acid were analyzed in tracheal aspirate, by commercial enzyme-linked immunosorbent assays (ELISA; R&D systems; Minneapolis, MN, USA), and uric acid levels were determined by the QuantiChromTM Uric Acid Assay Kit colorimetric assay (Bioassay Systems: DIUA-250). IL-6 and IL-1B have been previously described in association with BPD development [16, 17]. In addition we have tested new potential biomarkers in plasma and tracheal aspirate (IP-10, CCL5, ET-1 and uric acid) that could help predict the development of BPD [18–20].

### Statistical analysis

Considering that we aimed to identify high risk patients (moderate or severe BPD), all patients were classified into two groups: 1) No BPD group: absence of BPD and mild BPD; 2) BPD group: moderate/severe BPD and death. A comparative analysis of the characteristics of both groups was performed.

Although we obtained biomarkers data at 6 different time-points, for early BPD prediction we analyzed only data on day 7, this will be discussed in detail later.

The results are presented as mean and standard deviation if normally distributed or as median and interquartile range if not normally distributed. Differences in discrete variables were tested with Chi-squared or Fisher test. To compare means, Student’s t-test or ANOVA were used for normally distributed variables, and non-parametric tests if variables were not normally distributed. Correlations between quantitative variables were tested with Pearson or Spearman tests. A multivariate logistic regression, forward method, was performed to identify risk factors for BPD development by determining the odds ratio of both groups, no-BPD versus BPD, in relation to clinical, echocardiographic and analytic factors. All clinically relevant variables and those with a p-value <0.2 in the univariate model were included in the multivariate model. The sensitivity and specificity of the resulting models were evaluated by the AUC. A value of p<0.05 was considered significant. Stata version 14 was used for the statistical analysis.

## Results

**Fig 1:**
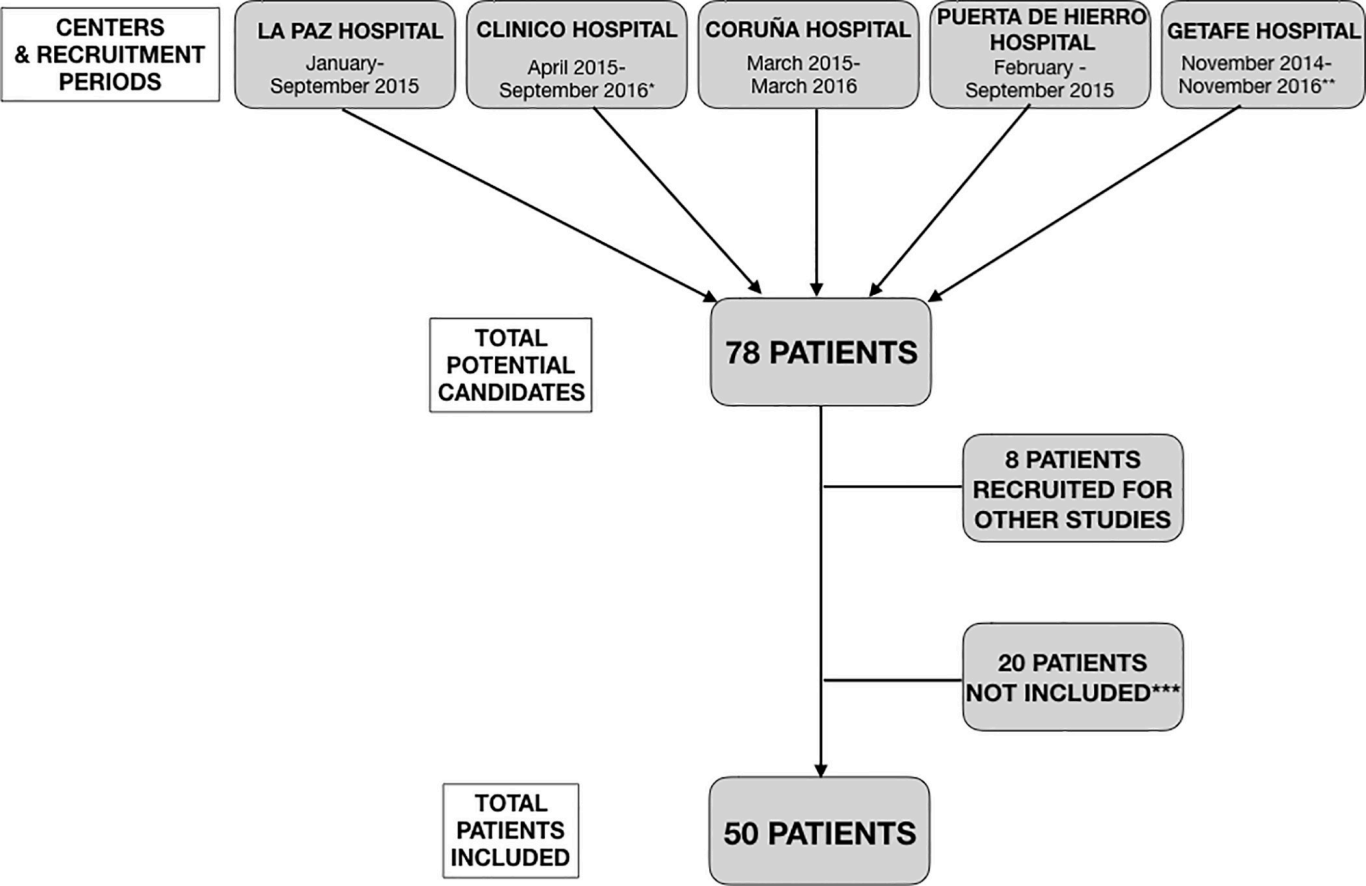
Flow chart showing the patient recruitment. Recruiting centers and recruitment periods are shown on the top row. Recruitment periods vary between hospitals due the existence of other studies recruiting the same type of patients. *One other study also aimed to recruit the same type of patients during this period. **During this two year period there were some interruptions in the recruitment due to circumstances not related with the study. ***It includes patients that did not meet inclusion criteria (3) and patients whose parents denied participation (11).

Fifty ELGAN were included between November 2014 and November 2016 (recruitment flow chart is available in Fig 1).

Three patients who died in the first four days of life were excluded of the analysis, considering that no data on day 7 was available.

Median gestational age was of 26 weeks (IQR 25–27). There were no patients with a gestational age under 24 weeks. In Spain, given the recommendations of the Spanish Neonatal Society, active resuscitation is performed over 24 weeks [21]. Under 23 weeks is rarely performed and between 23 and 24 weeks other criteria are take in account, such as the parent´s choice [21]. For this reason the number of preterm babies in Spain with a gestational age under 24 weeks is very low.

Mean birth weight was of 871 g (SD 161.0). All additional demographic characteristics, maternal and perinatal history, and patient outcomes are described in Table 2.

The global mortality in the study was of 20% (10 patients): 3 patients, died in the first 4 days; four after 28 days of life; and the other 3 patients died at 12, 15 and 25 days of life. These three patients had been on mechanical ventilation since birth, with persistent parenchymal lung disease and respiratory failure that could not be attributable to other neonatal morbidities, and therefore were considered to have high grade BPD (III (A)), a term recently proposed by Higgins et al [22].

The percentage of patients requiring mechanical ventilation (MV) for more than 7 days was significantly higher in the moderate/severe BPD group. Being on MV on day 7 was associated to the need of MV for a longer time (22.1 days (SD 9.95) versus 3.8 days (SD 5.26) (p<0.001)). Most notably, 81.8% of the patients who developed moderate or severe BPD were intubated at day 7, compared with 36% of the patients without BPD (p = 0.002).

**Fig 2:**
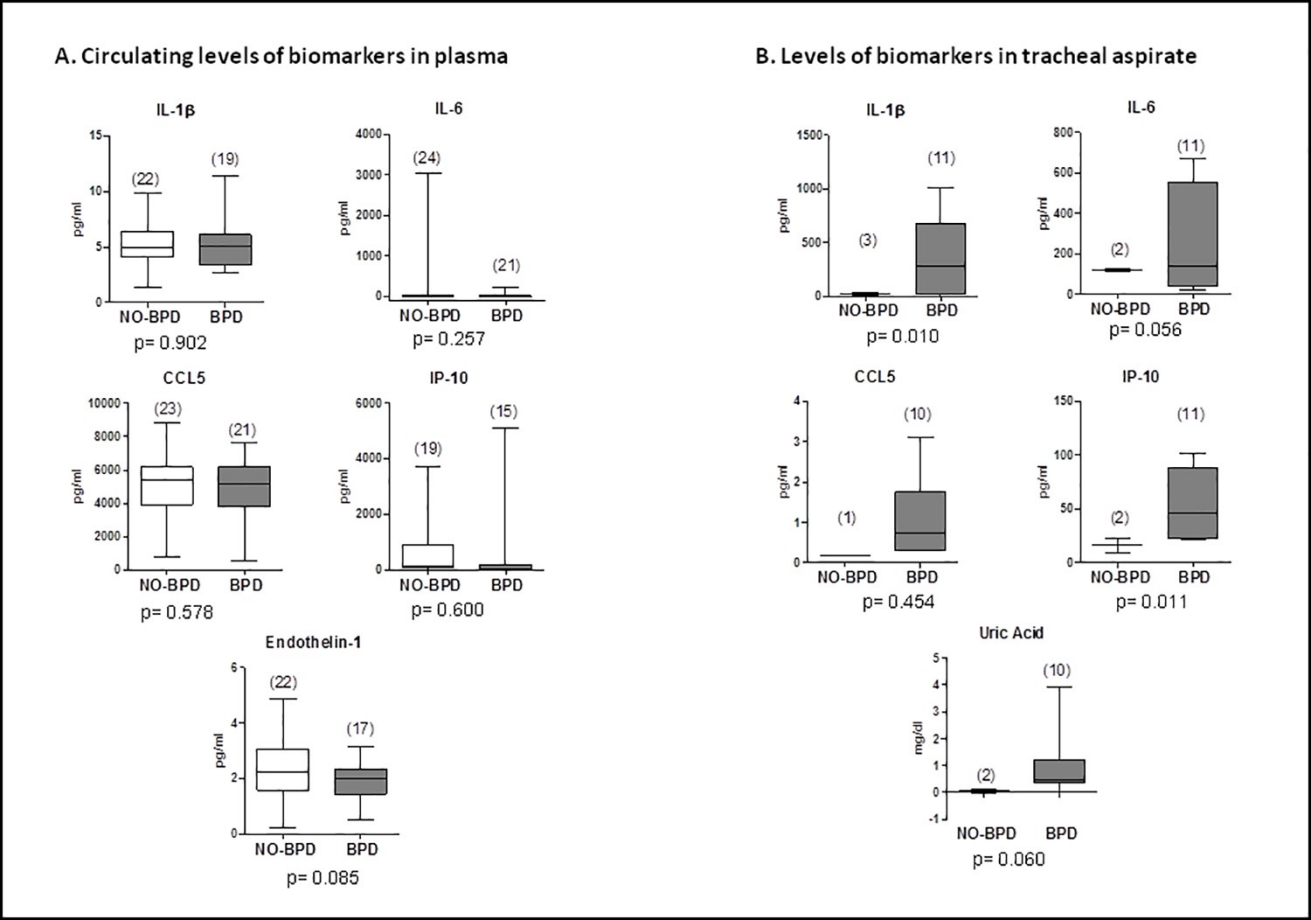
A. Levels of biomarkers in plasma of BPD (moderate/severe BPD or death) patients compared with no-BPD patients (no BPD or mild BPD) at day 7 of life. B. Comparison of the levels of biomarkers in bronchoalveolar lavage of intubated patients between the BPD and no-BPD group, at day 7 of life.

When comparing analytical biomarkers between both groups, we observed a significant increase in the levels of IL-1B, IL-6, IP-10 and uric acid in tracheal aspirate of moderate and severe BPD patients; whereas no significant differences were observed among plasma biomarkers (Fig 2). ET-1 was observed to be lower, at day 7, in newborns who developed moderate or severe BPD (p = 0.085).

After performing a univariate analysis (Table 3), we included variables with p<0.2 in the multivariate analysis (Table 4).

**Table 3:** General description of all patients and comparison of both study groups (no-BPD vs BPD).

	All Patients N = 47	no-BPD (no BPD/mild BPD) N = 25	BPD (moderate/severe BPD) N = 22	p value
Gender (Male)	57.4% (27)	52.0% (13)	63.6% (14)	0.421
Weight at birth (g)	871 (SD 161.0)	882 (SD 162.2)	859 (SD 162.6)	0.823
Gestational age (weeks)	26 (IQR 25–27)	26 (IQR 25–27)	26 (IQR 25–27)	0.894
Maternal smoking	8.5% (4)	9.1% (2)	9.5% (2)	0.961
Oligohydramnios	25.5% (12)	25% (6)	31.6% (6)	0.633
Chorioamnionitis	15.2% (7)	12% (3)	19% (4)	0.507
Prenatal steroids	89.4% (42)	92% (23)	90.5% (19)	0.855
Surfactant treatment	61.7% (29)	56% (14)	68.2% (15)	0.391
PDA (day 7 of life)	74.5% (22)	31.8% (15)	46.2% (7)	0.396
**PH at day 7 of life**	19.2% (9)	16% (4)	22.7% (5)	0.559
IVS flattening* at day 7 of life	54.6% (18)	45% (9)	69.2% (9)	**0.172**
Days on CPAP	20 (IQR 7–28)	17 (IQR 3–28)	20 (IQR 8–37)	0.299
Days on MV	4.3 (0.13–15.5)	1.4 (IQR 0–4.9)	18.3 (IQR 2.3–26)	**0.006**
**MV on day 7 of life**	27.7% (13)	36% (9)	81.8% (18)	**0.002**
**MV≥ 7 days**	57.5% (27)	16% (4)	40.9% (9)	**0.057**
Sepsis ≤ 7 days	8.5%(4)	4% (1)	13.6% (3)	0.237
**BPD** **- Mild** **- Moderate** **- Severe**	74.5% (35/47) 39% (14/35) 42% (15/35) 19% (6/35)			

BPD = bronchopulmonary dysplasia, CPAP = continuous positive airway pressure, PDA = patent ductus arteriosus, MV = mechanical ventilation, NEC = necrotizing enterocolitis. (*IVS types I-II or higher)

**Table 4:** Predictors of moderate or severe BPD (variables included in the multivariate analysis).

Variable	OR	SE	P>|z|	95%CI
**MV on day 7**	27.3	34.73	0.009	2.26–330.07
IVS flattening*	3.63	4.03	0.246	0.41–32.04
**ET-1 plasma on day 7**	0.34	0.22	0.096	0.10–1.20

MV = mechanical ventilation, IVS = interventricular septum, ET-1 = endothelin 1. (*IVS types I-II or higher)

When analyzing echocardiographic variables, in the univariate analysis, we observed that patients with PDA or with PH at day 7 had a greater risk of moderate or severe BPD. In the multivariate analysis only interventricular septum (IVS) flattening, as indirect sign of PH, was significant and therefore included in the model. All other echocardiographic variables such as atrial septum defect, right ventricle dilatation or left ventricle ejection fraction, were not related with moderate or severe BPD outcome.

Among these results (Table 4) we observe that, on day 7, the combination of being on MV and having echocardiographic signs of PH, interventricular septum (IVS) flattening (septum types I-II or higher), can predict moderate or severe BPD with a sensitivity of 61.5% and specificity of 85% (AUC 0.773). The addition of plasma ET-1 increased the sensitivity to 63.6% and specificity up to 94.4% (AUC 0.861). An increase in ET-1 levels was associated with a decrease in BPD risk, although it did not reach statistical significance probably due to the small sample size.

## Discussion

Our study shows that a very simple predictive model composed of 2 variables (MV and echocardiographic signs of PH), is able to predict moderate or severe BPD, with a reasonable sensibility and specificity. We also found that the addition of plasma ET-1 (a pathway not yet well explored in BPD), to that model, improved the predictive value.

Early BPD diagnosis has been a recurrent research goal for the past decades. Numerous studies have analyzed clinical and analytical biomarkers to identify the optimal BPD prediction strategy [17, 23, 24]. Unfortunately, no single biomarker or combination of biomarkers with a good predictive value has been found. A great number of predictive models using clinical variables have been developed, but none has been able to determine which high-risk patients will eventually develop BPD [13, 14, 24]. Most predictive models agree on a number of BPD risk factors, such as birth weight, GA, chorioamnionitis, preeclampsia, respiratory parameters, etc. [13]. These known risk factors increase neonatologists’ awareness of the potential risk of BPD in selected patients, but are still not able to identify only patients with high risk of moderate or severe BPD and tend to overestimate this risk. This makes it difficult to implement early interventions for selected patients who will, with high probability, develop the worst grades of the disease.

Another issue under discussion is the optimal time-point for risk determination of BPD. Some models have tried to stratify the risk in the first week of life or even at birth, considering mostly prenatal factors [24]. We consider that day 7 is an optimal time-point in the patients’ course to determine the risk of moderate or severe BPD. Many ELGAN can have a favorable evolution during the first days of life but can deteriorate afterwards, due to postnatal factors (MV, PDA, infections etc.) [12]. During the first week of life the lung is starting to deteriorate but there is still a healing potential in the lung tissue [22]. Therefore day 7 is a good time-point to start preventive therapies. Afterwards, the lung damage will perpetuate and the lesions might not be reversible, especially when they have evolved to fibrotic stages [22, 25]. In addition, when having in mind regenerative medicine as preventive treatment for BPD, this will require several days for preparation (up to a week). Therefore we should know in advance which patients are at risk of moderate or severe BPD in order to have time to prepare and initiate the therapy in the first two weeks of life [25].

After exploring clinical, echocardiographic and analytical variables, alone and in combination, we establish that the combination of MV dependency; echocardiographic signs of PH (IVS flattening); and the elevation of certain biomarkers on day 7 of life can predict the risk of developing moderate or severe BPD.

MV is a harmful factor responsible for lung injury [26]. Although there have been great improvements in pulmonary care towards more protective ventilatory strategies, time on MV continues to be the main risk factor for BPD [13]. BPD has been related to the administration of high oxygen concentrations and to elevated airway pressures [23, 24]. However, MV itself is an independent risk factor for BPD, regardless of ventilatory settings, considering that the risk of lung infection and inflammation are also increased. In our cohort, not only did patients who were on MV for more time have a greater risk of BPD, but we defined a cut-off point beyond which MV itself becomes a risk factor for BPD. We estimated that above 7 days of life, ELGAN on MV have a 27-fold increased risk of moderate or severe BPD (Table 4). Similar results were described by Laughon et al. who consider that the two main risk factors for BPD are GA and MV [13].

An echocardiographic study on day 7 of life established that patients with PDA or with PH at that time-point had a greater risk of moderate or severe BPD. Therefore, to increase the predictive power of MV as a risk factor, we selected the echocardiographic parameter of IVS flattening [8]. Patients with IVS types I-II or higher (indirect sign of PH) have a 3.6-times greater risk of developing moderate or severe BPD (Table 4). With these two parameters (MV and IVS flattening) on day 7 of life, we can predict moderate or severe BPD with a sensitivity of 61.5% and specificity of 85% (AUC 0.773). Recently, it has been described that the presence of established PH on echocardiography between the 3rd and 14th day of life is associated with decreased in-hospital survival and moderate-to-severe BPD, which reinforces the importance of PH as a predictive factor [8, 27].

We also analyzed plasma and tracheal aspirate levels of biomarkers associated with the development of MV-induced lung injury, BPD and/or PH. Inflammatory biomarkers, like IL-1B and IL-6, are associated with a worst outcome in patients with PH or adult respiratory distress syndrome [28, 29]. Circulating levels of IP-10 correlate with pulmonary hemodynamics in patients with PH and may underlie the recently uncovered link between interferon and PH [18]. Uric acid, produced at large amounts from injured tissue, is released in the lung as response to mechanical ventilation; it triggers lung inflammation and fibrosis and induces pulmonary endothelial dysfunction [18–20]. Although no significant differences were found on day 7 in the circulating levels of these biomarkers, our results suggest that the levels of IL-1Β, IL-6, IP-10 and uric acid are increased in tracheal aspirates following lung injury and could be related with the latter outcome of BPD (Fig 1). IP-10, uric acid and CCL5 are associated with lung disease and pulmonary hypertension. After testing the relationship of these molecules with BPD prediction, we observed that IP-10 and uric acid are potentially related with the development of moderate or severe BPD (Fig 1). CCL5 did not reach statistical significance (Fig 1). The absence of correlation that we observe between tracheal aspirate and blood levels of these biomarkers, can be due to a local increase of these molecules. However, because these samples were only available in intubated patients, the small number of samples included in our study not only precludes drawing any firm conclusion but disqualify these variables to be introduced in the regression model.

ET-1 is a peptide with vasoconstriction properties secreted by endothelial cells. In animal models, ET-1 is increased in PH and plays an important role in the progression to lung fibrosis [30]. Kuo et al. observed higher levels of tracheal ET-1 in respiratory distress syndrome but no differences the ET-1 levels in relation to BPD [31]. Andersson et al. explored ET-1 levels in a group of preterm infants and observed that high levels of ET-1 in the airways related with less severe respiratory distress in the early postnatal period [32]. They postulate that ET-1, in addition to a pro-inflammatory and pro-fibrotic effect, stimulates the secretion of surfactant in the lung, which can improve lung development in the long term [32]. Although the pathogenic role of ET-1 in BPD is unclear and higher levels of ET-1 were expected in the moderate/severe BPD group, in our patients, we observed that lower levels of ET-1 in plasma were a risk factor, increasing the predictive value for the diagnosis of moderate or severe BPD. A plausible explanation would be the one suggested by Andersson et al. who consider that ET-1 stimulates the production of alveolar surfactant in the preterm lung [32]. This would result in an improved lung development in patients with early high levels of ET-1 and an adverse outcome in those with low plasma levels. In addition, ET-1 would also increase once BPD and fibrosis are established. However, the role of ET-1 in the premature lung needs further research, and our results regarding plasma ET-1 levels will have to be confirmed and validated in a larger sample.

When comparing the two models (MV+IVS with MV+IVS+ET-1), we achieved a small increase in sensitivity and specificity with the addition of ET-1 to the model. Although the low levels of ET-1 in high risk moderate/severe BPD patients is one of the most interesting findings of this research we consider the use of the two-variable model (MV + IVS) more feasible in the clinical setting. The role of ET-1 in BPD is not completely understood and further research should be performed before using it in the clinical practice. Also the determination of ET-1 still requires special laboratory determination techniques, which makes it hard to use ET-1 on a daily bases.

Early BPD diagnosis is one of the main goals of neonatologists. Knowing which patients are at greater risk of developing BPD in the first week of life can make a difference in the outcome of moderate or severe BPD, by applying preventive strategies. Therefore, for the neonatologist it is very important to detect, at the patient’s bedside, high risk patients. With the model we propose, if the patient is on mechanical ventilation on day 7 of life, and he/she has indirect signs of pulmonary hypertension [8], this patient should be considered a high risk patient in which preventive strategies should be applied. It is a very simple model and very easy to achieve at neonatal intensive care units.

The findings of this study can be used to guide the design of clinical trials testing preventive strategies for BPD, such as regenerative therapies. For this goal it is important to identify only patients at risk of moderate and severe BPD, who have the worst outcomes and can benefit from preventive strategies.

The strengths of this study are its prospective nature in a population of babies at the highest risk of developing BPD (ELGAN) and the possibility it offers of determining the risk of BPD at a very early stage and at the patient’s bedside by a simple method: assessing the need for MV on day 7 and performing a routine echocardiographic evaluation on that day. In addition, this study is the first to explore the role of plasma ET-1 levels as a biomarker for predicting BPD in ELGAN.

The main limitation of the study is the small number of patients, which allows us to explore these novel molecular biomarkers but not to achieve conclusions on the role of ET-1 in BPD. Another limitation is the decreased number of tracheal aspirate samples, considering that tracheal aspirates were obtained only from intubated patients.

In summary, the combination of clinical and echocardiographic variables is a valuable tool for determining the risk of moderate or severe BPD. Future research on BPD prediction should be oriented to explore the potential role of ET-1, and also of miRNA determinations, which could be useful not only for BPD prediction, but also to elucidate pathogenic mechanisms and potential therapeutic targets in BPD.
